# Growth of a common planktonic diatom quantified using solid medium culturing

**DOI:** 10.1038/s41598-018-28129-y

**Published:** 2018-06-27

**Authors:** Olga Kourtchenko, Tuomas Rajala, Anna Godhe

**Affiliations:** 1 University of Gothenburg, Department of Marine Sciences, Gothenburg, SE 405 30 Sweden; 2 University College London, Department of Statistical Sciences, London, WC1E 6BT United Kingdom; 3 Chalmers University of Technology, Department of Mathematical Statistics, Gothenburg, SE 412 96 Sweden

## Abstract

The ability to grow on solid culture medium is a pre-requisite for a successful microbial genetic model organism. *Skeletonema marinoi*, a bloom-forming, planktonic marine microalga, is widely used in ecological, evolutionary and population genetics studies. We have tested and confirmed the ability of this common organism to grow on solid culture medium (agar) under experimentally manipulated conditions. We established a protocol for quantifying growth characteristics – length of lag phase, growth rate, maximum biomass yield – on agar medium. The procedure was tested under experimental treatments and the resulting growth changes correlated with those observed in standard liquid culture. The ability to grow on solid medium broadens the use of *S*. *marinoi* as a molecular model, where agar is routinely used for various purposes (growth, selection, storage); and the possibility to quantify colony growth opens the way for high throughput, automated, or semi-automated phenotyping solutions.

## Introduction

Agar-based solid medium is widely used in molecular and microbiology for various purposes, including cell culturing, screening, selection, and storage. In recent years, it has become recognized for its power in quantitative microbial phenotyping, allowing for scaling of phenotyping throughput to unprecedented levels^1,2^. The existing technology in this context caters primarily heterotrophic microorganisms (e.g. bacteria, fungi) that utilize the energy of an organic carbon source supplied in the medium. In contrast, few solid medium-based technologies are employed for microbial phototrophs (organisms that rely on the energy of the light to fix inorganic carbon) that are not used as the molecular model species (e.g. *Clamydomonas reinhardtii*, *Synechocystis sp*., *Phaeodactylum tricornutum*); and none of these technologies have been applied to quantitative growth phenotyping^3,4^. The requirement for controlled light intensities increases the cost of automated phenotyping solutions, and adds another level of complexity to this already non-trivial task.

*Skeletonema marinoi* Sarno & Zingone is a unicellular, chain-forming diatom, common in temperate coastal waters worldwide, and often dominating spring algal blooms^5^. It is a widely used species in phytoplankton ecology, evolution and population genetics^6–8^; and it is currently being developed as a model for molecular studies and functional genomics^9–11^. The ability to grow on solid medium is a pre-requisite for many of the molecular and screening techniques.

*Skeletonema*, like the majority of planktonic microalgae, is characterized by extremely high levels of clonal diversity and enormous population sizes, especially during blooms^12,13^. In addition, due to its ability to form resting stages, *Skeletonema* maintains an archive of genotypes buried in the sediments, dating back hundreds of years^14^. Both vegetative and resting cells are easily isolated from the wild and maintained under laboratory conditions. Consequently, the ability to phenotype large numbers of individuals is an urgent necessity in this field, where many studies are still done on relatively small sets of strains, mainly due to technical limitations. Recently, advances have been made in the direction of plate-based phenotyping in liquid culture medium^15–17^. Since solid medium based phenomics promise a higher degree of throughput, we have tested this avenue for our species of interest.

In the present study we tested the ability of *S*. *marinoi* to grow on agar-solidified culture medium. Moreover, we evaluated the potential of such culturing technique for quantitative phenotyping of *S*. *marinoi* growth under manipulated experimental conditions. To our knowledge, this is the first attempt to quantify growth of a pelagic microalga on solid medium.

## Results

### Quantification of *Skeletonema marinoi* growth on solid medium

#### General observations

**Figure 1:**
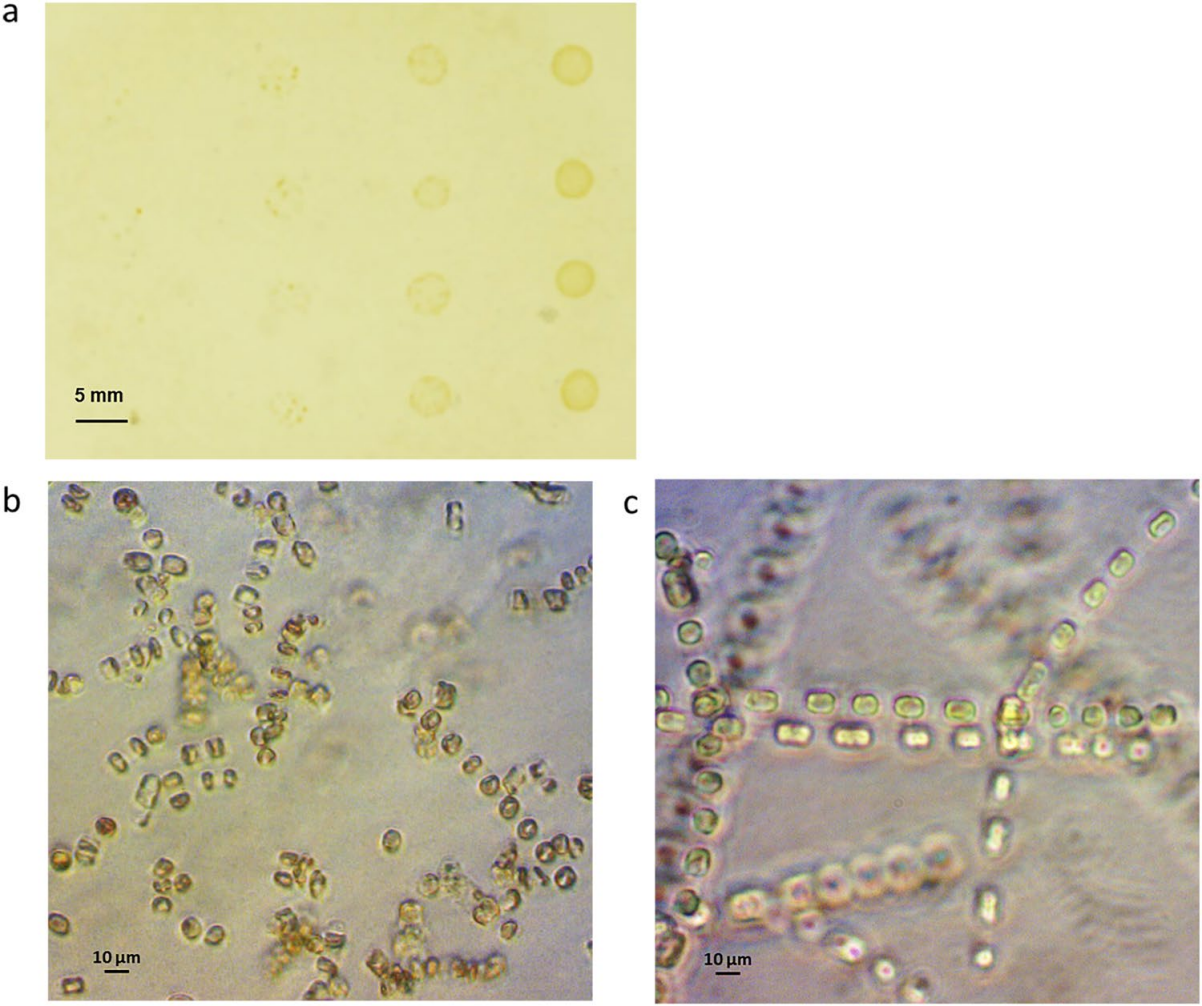
Colony and cell-chain morphology of agar-cultured *S*. *marinoi*. The appearance of *S*. *marinoi* colonies on a surface of an agar-based solid medium, plated in quadruplicates in four 10-fold serial dilution steps (40–40 000 cells per colony), after one week on culturing at 10 °C (26 practical salinity units (psu); light intensity 50 µmol photons m^−2^ s^−1^) (**a**). The morphological appearance of *S*. *marinoi* (strain RO5AC) cells and chains under 400× magnification of an inverted light microscope after one week of culturing on solid medium (**b**), or standard liquid medium (**c**).

We began by establishing the amenability of *S*. *marinoi* to proliferation on a surface of solid culture medium, such as agar. To this end, we plated a fast-growing laboratory strain, RO5AC at different starting cell densities (four to 40 000 cells per colony). We observed the appearance of visible colonies after a week of culturing at 10 °C for all of the dilutions down to 40 starting cells per colony (Fig. 1a). The agar-grown *Skeletonema* cells appeared morphologically similar to the cells cultured in liquid medium at the 400× magnification of an inverted light microscope (Fig. 1b,c). Notably, we observed that chain formation was not impeded by the growth on agar plates; however, the overall chain length appeared to be reduced in the agar-cultured sample (Fig. 1b,c).

#### Integrated pixel density - a proxy for colony growth

**Figure 2:**
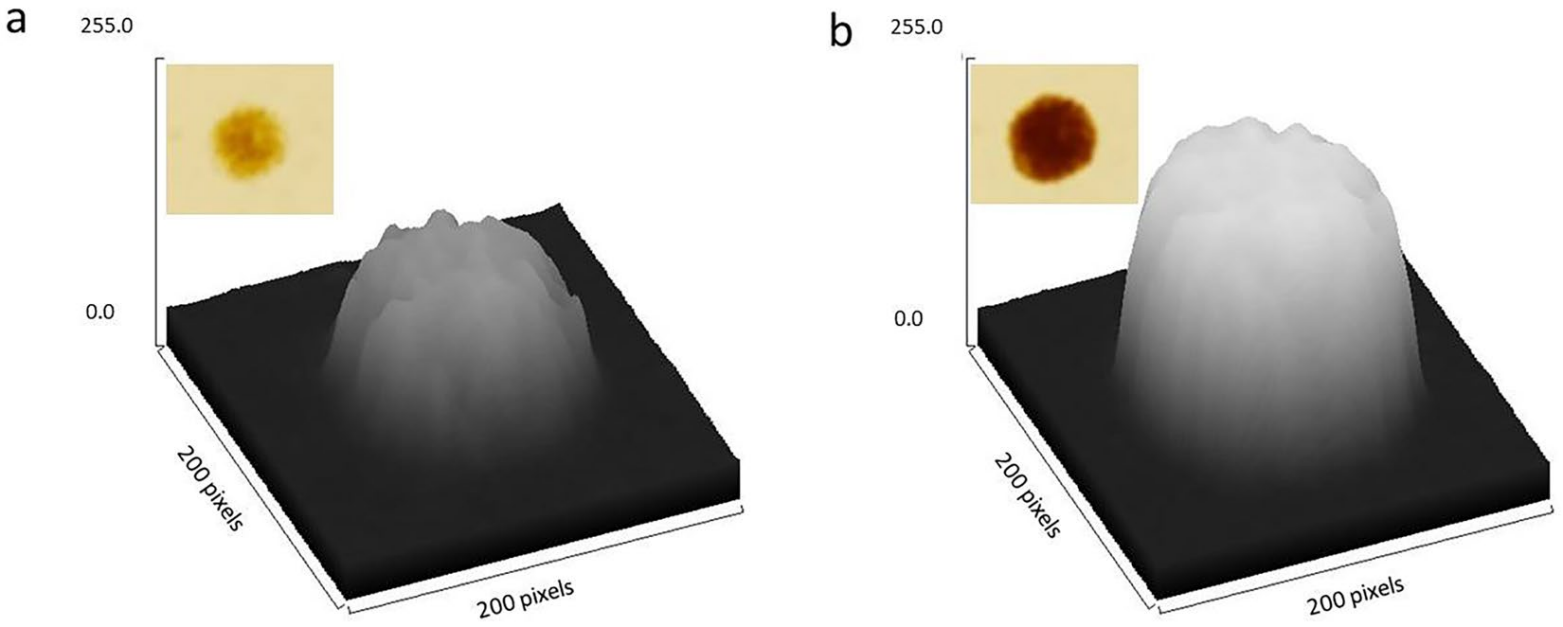
Graphical representation of a colony in three dimensions. z-axis represents the intensity values for each pixel within the colony area (x- and y-axis). The insert image shows the appearance of the corresponding *S*. *marinoi* colony in the exponential (**a**) and in the stationary (**b**) growth phases.

To be able to quantify growth of a *S*. *marinoi* colony on solid medium, we needed to find a suitable proxy for the cell biomass. We observed that growing colonies were first becoming darker in color, followed by an increase in their surface area (Fig. 2a,b). Specifically, the color change, measured as the change in average pixel value of the colony image, increased two to four fold, while the colony area incremented about 2.5 times of the initial value (5 mm^2^). Assuming that the starting cell inoculum (approximately 20 000 cells per colony) would be evenly spread over the colony area, about 80% of the colony surface would be initially free of cells. Cell proliferation would then result in increasing cell density (perceived as darker color) both within the colony area and outward. In order to take into account this within-colony growth, we used the sum of colony’s pixel values, or integrated pixel density, to represent cell biomass (Fig. 2a,b).

### Growth phenotypes of *Skeletonema marinoi* under temperature and salinity treatments

Once the quantification procedure for solid medium cultures was established, we tested the performance of this method and its suitability for quantitative phenotyping, using salinity and temperature manipulated conditions.

#### Growth phenotypes under liquid medium culturing

**Figure 3:**
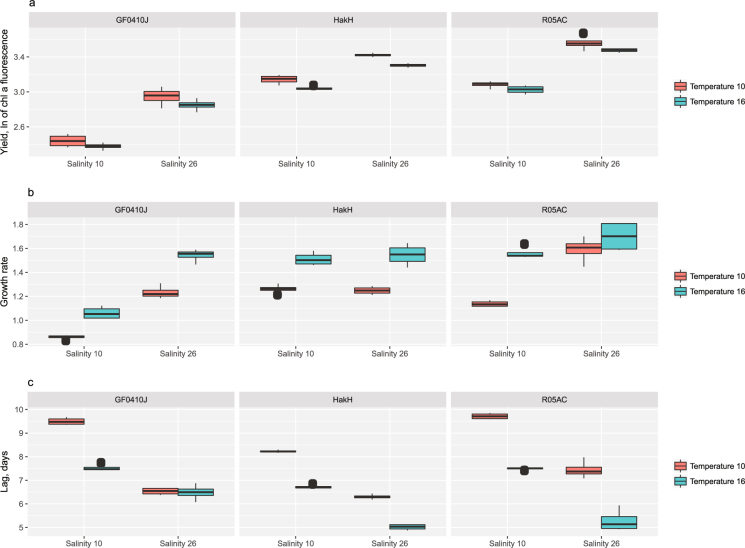
Quantitative phenotypes in liquid culture medium. Growth responses of three *S*. *marinoi* strains (GF0410J, HakH, RO5AC) to the treatment conditions (salinity 10 psu vs. 26 psu–x-axis; temperature 10 °C vs. 16 °C-red and blue boxes) in liquid f/2 + Si medium: maximum yield (ln-transformed chlorophyll a fluorescence values, **a**), growth rate (**b**), lag (days, **c**) (n = 4).

In order to establish a comparison baseline for the solid medium phenotypes, we first measured growth of the three test strains (GF0410J, HakH, and RO5AC) in liquid medium under the two temperature and salinity regimes. We observed clear quantitative differences between the tested strains in response to the treatments, as well as general treatment responses common for the three tested strains (Fig. 3a–c). Specifically, low salinity (non-native) resulted in a lower maximum yield (effect size between −0.3 and −0.5 ± 0.08, depending on the strain; p-value 4.3e-15), and this effect was independent of the temperature treatment (Fig. 3a). Higher temperature, as expected, resulted in faster growth (mean effect size 0.2 ± 0.03; p-value 4.6e-06), this effect varied quantitatively depending on salinity and strain (Fig. 3b). Finally, the lag-time phenotype was remarkably similar between the tested strains, where increase in temperature and salinity resulted in shorter growth delay (Fig. 3c). GF0410J strain was an exception in this regard, where the lag time at higher salinity was not affected by the temperature (Fig. 3c).

#### Correlation between growth parameters obtained from agar colonies and liquid cultures

**Figure 4:**
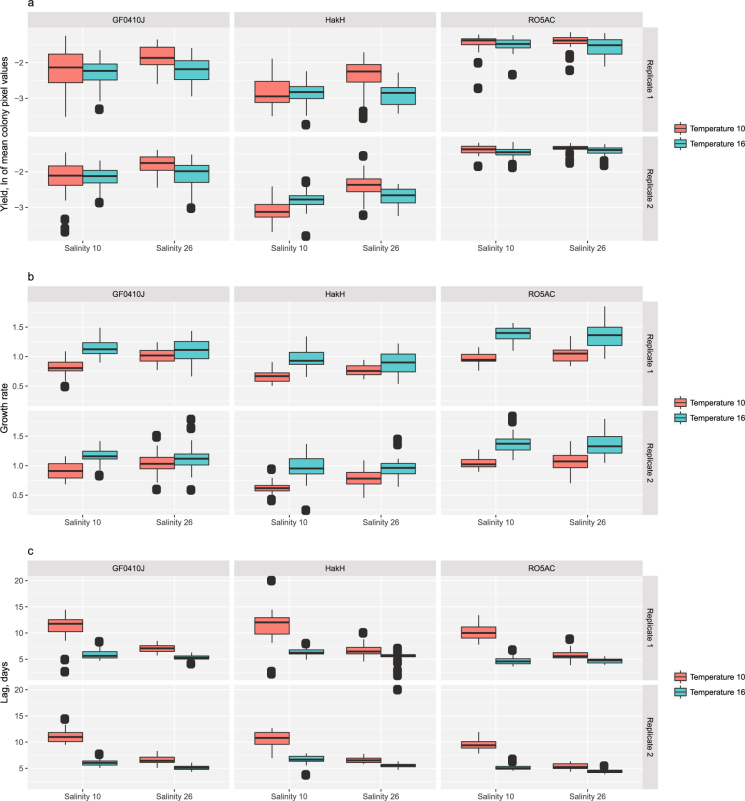
Quantitative phenotypes on solid culture medium (agar). Growth responses of three *S*. *marinoi* strains (GF0410J, HakH, RO5AC) to the treatment conditions (salinity 10 psu vs. 26 psu - x-axis; temperature 10 °C vs. 16 °C- red and blue boxes) on agar f/2 + Si medium: maximum yield (ln-transformed mean pixel values, **a**), growth rate (**b**), lag (days, **c**) (n = 32*). The results for both replicate plates are shown. * - the actual number of replicates used in the analysis was lower than 32 for some of the treatment plates due to the removal of dead colonies, see Methods: Statistical analysis.

Parallel to the liquid medium growth assays, we performed the same experiments on solid medium and contrasted the extracted quantitative phenotypes for two media types. As the result, we observed a partial overlap in growth phenotypes of solid and liquid media-cultured test strains in response to salinity and temperature. Specifically, the average (for the three tests strains) effect size (−0.38 ± 0.11; p-value 3e-10) of reduced max yield in lower salinity was similar between the two growth medium conditions (liquid and solid). This effect on agar medium was apparent in two out of three test strains (GF0410J and HakH) at the low temperature treatment (10 °C; Fig. 4a). The positive effect of increased temperature on the strains’ growth rate was also observed in the agar-grown cultures, albeit less pronounced than in the liquid medium (Fig. 4b). The temperature effect on growth rate was significant in the low salinity treatment, with the exception of the fast-growing RO5AC strain, where this was true for both salinity regimes (Figs 3b and 4b). Agar-grown cultures displayed a similar pattern of reduced lag time in response to increased temperature and salinity as observed in liquid medium (Figs 3c and 4c). In contrast to the liquid-grown cultures, the temperature effect on the lag time was stronger in low salinity treatment in all three strains (Fig. 4c).

**Figure 5:**
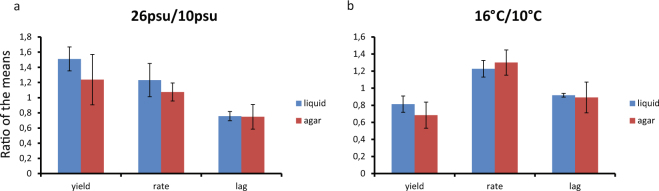
Generalized treatment effects on growth phenotypes. Treatment effects on yield, rate, and lag are summarized into one value per phenotype by taking the mean of the corresponding values for all strains. The bars represent the ratio between these means for the two modes of the treatment (e.g. 10 °C and 16 °C for the temperature treatment). Phenotype ratios in salinity treatment (**a**), and in temperature treatment (**b**). Blue bars - liquid culture conditions, red bars - agar culture conditions. Error bars represent standard deviation from the mean for each group of values (n = 6).

To summarize the tested treatment effects on *S*. *marinoi* we took a mean of individual strains’ responses (yield, rate, lag) grouped by either salinity or temperature. We then compared the corresponding means (e.g. mean rate at 16 °C vs. mean rate at 10 °C) for each of the growth phenotypes by taking a ratio of the means corresponding to the two treatment conditions (16 °C vs. 10 °C, and 26psu vs. 10psu) in each treatment group (Fig. 5). The results show a general pattern of shortened lag time and increased growth rate in response to increased temperature (Fig. 5b) in both types of medium (liquid and solid). In contrast, the general response to higher salinity of increased growth rate and yield, observed in liquid medium, was not noticeable on agar. However, the shortening of the lag time at higher salinity was consistent between the two types of growth media (Fig. 5a).

#### Variability in the solid medium growth data

In order to assess the precision of the method, we quantified the variability in our agar data both within a plate (32 replicate colonies per strain), and between plates (two replicate plates per treatment).

We addressed potential spatial biases of our agar-culturing method by arranging the 32 replicate colonies across the plate in a regular pattern (see Supplementary Fig. S1). When mapping the resulting yield and rate values for each colony position, we noted no systematic variation in these, corresponding to the colony’s position on the plate (see Supplementary Fig. S2).

**Figure 6:**
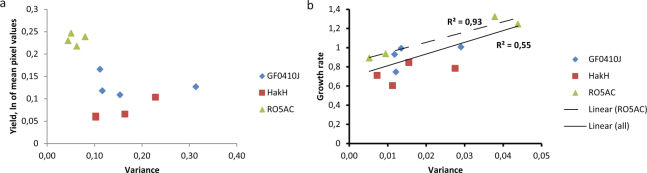
Relationship between a phenotype estimate and its associated variance. Mean (n = 32*) strain and treatment-wise yield (ln-transformed mean pixel values, **a**), or growth rate (**b**) values (y-axis) are plotted against the corresponding variance values (x-axis). Linear regression trend line and the corresponding correlation coefficient (r2) are given for the RO5AC strain alone (dashed line) and for the whole data set (solid line). * - the actual number of replicates used in the analysis was lower than 32 for some of the treatment plates due to the removal of dead colonies, see Methods: Statistical analysis.

Within plates we did, however, observe lower variance for RO5AC colonies in all treatments, parallel with higher overall cell densities (higher yield) of this strain compared to the others (Fig. 6a). GF0410J and HakH, attaining relatively lower yield, had higher values and wider spread of the associated variances (Fig. 6a). Conversely, the rate estimates varied more for the strains and treatments exhibiting faster relative growth (r2 = 0.54, Fig. 6b)). Specifically, for the RO5AC strain the correlation coefficient between the mean rate and the associated variance was at 0.92 (Fig. 6b). The overall coefficients of variance for both yield and rate values averaged at around 15% (8–25%, depending on the strain and treatment).

**Figure 7:**
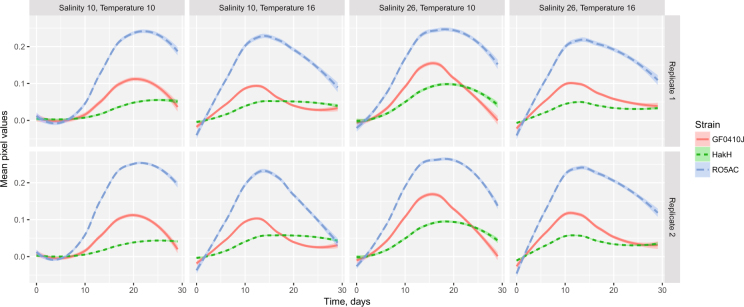
*S*. *marinoi* growth curves on replicate agar plates. Each curve represents an average smoothed time series of one of the three *S*. *marinoi* strains (GF0410J - red, HakH - green, RO5AC - blue) under the treatment conditions on agar medium supplemented with f/2+ Si medium. y-axis shows mean pixel values of each colony averaged across within-plate strain replicates (n = 32*). * - the actual number of replicates used in the analysis was lower than 32 for some of the treatment plates due to the removal of dead colonies, see Methods: Statistical analysis.

To assess the variability in growth estimates between plates, we modeled the phenotypes with a linear model:

Growth phenotype ∼ Salinity * Temperature * Strain + Replicate. Analysis of each term’s contribution (ANOVA and AIC) indicated no significant effect of the “Replicate” term (see Supplementary Table S1). Moreover, the average strain growth curves were nearly indistinguishable between the replicate plates (Fig. 7).

Taken together, the results suggest an agreement between replicate assays, but also the need for within-plate replication. The degree of such replication (the minimum number of within-plate replicates) depends on the organism, strain, treatment, and the change in response due to treatment or effect size. For example, in our study the effect size due to increased temperature, all else being equal, was on average 10% in yield and 30% in rate and lag. With the observed levels of variation (pooling the replicated experiments), we can approximate the required number of samples for detecting such effects sizes. If we expect to see observed baseline phenotypes at 10 °C temperature treatment as we had now, high certainty (i.e. 90% statistical power) of detecting a change of, for instance, 20% can be achieved with as low as 10 replicates per plate (e.g. lag), or require as many as 35–40 replicates per plate (e.g. yield of GF0410J, rate of HakH). An overall median sample size requirement in this set-up is around 18 replicates per plate. Note that in the liquid medium, due to lower variability, similar number of samples would be enough for detecting effect sizes as low as 5%.

## Discussion

We have assessed the ability of *S*. *marinoi*, a common phytoplankton species, to grow under solid medium culturing conditions. Furthermore, we developed a methodology to quantify three important growth characteristics under such conditions, experimentally tested and compared these to growth responses in conventional liquid culture.

The few reports, describing or mentioning the use of solid medium applications for planktonic or benthic diatoms, rely on lower strength agar solution (1.5% and lower), or on modifying the agar surface via introducing grooves and channels, which permit algal growth^4,18–20^. In our study, *S*. *marinoi* successfully proliferated on a standard, unaltered agar-solidified medium, maintaining its chain-forming ability and the general morphology of liquid-cultured cells. These findings open the opportunity for a more labor- and cost-effective alternative to maintaining a large number of *Skeletonema strains*, and, potentially, other microalgal strains in culture collections. An example of this is the ongoing *S*. *marinoi* transformation project^10^, where mutant generation, screening, assay, and maintenance are facilitated by the use of solid culture medium.

Solid medium is a powerful tool in the arsenal of quantitative microbial phenomics, as it allows for high precision, accuracy and throughput^1^. The field of phytoplankton ecology and evolution has obvious gains from access to such technologies, where low (manual cell count) and medium (automated cell count, or *in vivo* chlorophyll fluorescence measurements) throughput methods currently prevail^15,17,21,22^. In the present study we have taken the first steps in applying and evaluating growth quantification on solid medium for a planktonic, ecologically relevant organism, *S*. *marinoi*. One outcome of this work is a method pipeline adapted for a photoautrophic (requiring light as the energy source for growth) microorganism. The described method has the potential for scaling and for automation of data acquisition and image processing.

We focused our analysis of solid medium *S*. *marinoi* cultures on the three parameters that are traditionally extracted from the microbial growth curves (lag time, growth rate and yield) and used them to approximate microbial fitness characteristics^23–25^. We were able to successfully determine all three growth parameters from the image data, and found them (for the most parts) in agreement with the same parameters produced by the liquid cultures. Specifically, both medium types (liquid and solid) supported faster growth (shorter lag time and higher growth rate) of all tested *S*. *marinoi* strains in response to increased temperature. Notably, even the absolute values for the lag and rate parameters agreed between the two tested medium types.

The similarity in the observed lag times between liquid cultures and agar colonies argues in favor of the selected started inoculum for the solid medium experiment. The starting cell concentration for the liquid culture was selected based on our previous experience with this organism^17^. We expected lower sensitivity of our agar colony phenotyping method, compared to the chlorophyll *a* fluorescence measurements used for cell suspensions. Furthermore, we assumed that exposing *Skeletonema* cells to agar surface conditions would in itself impose a longer adaptation time, when compared to the standard liquid culture conditions. Thus, in order to minimize the so-called inoculation lag we chose a higher initial cell density of agar colonies^25^. The lack of, or small differences in lag time between liquid and agar-cultured cells suggests that the recorded phenotypes likely reflect true physiological difference rather than a technical artefact.

The mean growth rates were also in the same range for the both media types, and corresponded to about one cell doubling per day - the average expected cell division rate of *S*. *marinoi* under standard laboratory conditions. Thus, despite using different proxies for the cell number (chlorophyll *a* fluorescence in liquid culture vs. mean pixel values for agar colonies), the rate estimates in both cases appeared to match each other and the documented norm obtained via manual cell counts^7^.

The maximum yield values, however, are not as readily comparable under the current set up; only the relative changes in a strain’s yield in response to treatment could be compared. For the purpose of this study, such level of comparison was sufficient, and, indeed, we recorded similar response patterns in cultures yield due to temperature treatment in the two media types. Our method could be further developed to include a standard function for converting pixel values to cell number, bio-volume, or another desired metric to represent biomass. This could be a useful tool for investigating the effects of solid medium culturing conditions on the strain productivity, for example. A standard function would also inform about the detection and quantification range of the method, the linearity and non-linearity between the two types of measurements (e.g., pixel value and cell number). However, as the conversion is not exact and carries uncertainties, changing the values from one metric to another would increase the overall error.

Surface growth for an organism natively existing in suspension seems like a foreign concept, based on which we expected to find differences in growth parameters of agar colonies compared to the liquid culture. Our results revealed this distinction to be *S*. *marinoi* growth response to salinity. Growth rates of agar-cultured cells were lower than those in the corresponding liquid cultures. This observation likely reflects the distinctiveness in physio-chemical conditions imposed by the two culturing modes on the *S*. *marinoi* cells. More importantly, however, the positive effect of higher salinity on the growth parameters, significant in our liquid medium experiment, was virtually absent on solid medium. This discrepancy hints at an imbalance in nutrient availability, experienced by the *S*. *marinoi* cells on agar surface and in liquid suspension; and this imbalance is either driven by, or related to the water salinity. One obvious factor that could be contributing to the described differences, is the greater contact with the gas phase for agar surface conditions: it facilitates the exchange of both carbon dioxide (phytoplankton’s carbon source) and oxygen, as well as presumably lowers the osmotic pressure that the cells experience^26^.

Despite the commonality in growth response patterns to treatment of the tested *S*. *marinoi* strains, they behaved differently quantitatively (some strains performed better than others) in the same medium and between liquid and solid medium types. Specifically, GF0410J was the slowest-growing and attained lowest final cell density in liquid cultures, whereas on agar HakH displayed a similar response instead. These observations likely reflect strain-specific abilities to tolerate solid medium culturing conditions and highlight the underlying genetic differences between them.

All known microbial micro-cultivation growth and phenotyping techniques, using liquid or solid media, suffer from biases imposed by the so-called edge effect, where the growth characteristics of the individuals are affected by their position on the plate relative to the edge^16,27^. For the techniques involving solid medium, the situation is further complicated by the neighboring colony-imposed effects, which scale with the resolution of the method^1^. No spatial biases were revealed at the level of resolution (total number of colonies per agar surface area) used in our study, despite the high degree of replication across the plate. Supposedly, such biases could become more relevant for studies using higher in-plate colony densities, aiming at a higher throughput.

Finally, we report higher statistical variability between replicate samples in the solid medium setup compared to the liquid cultivation, equaling roughly to order five in terms of coefficient of variation. Clearly, the procedures for liquid medium are well established and future improvements in the image processing and phenotyping pipeline should reduce the difference, increasing statistical power and reducing required in-plate replication.

In conclusion, our study demonstrated the ability of *S*. *marinoi*, an organism typically occurring suspended in a water column, to proliferate on a surface of solid medium, such as agar. Furthermore, we were able to quantify colony growth of this species by developing a data collection and processing pipeline. The pipeline was tested under an experimental setup and the resulting colony growth characteristics: lag time, growth rate and yield, were shown to agree, in most cases, with those obtained in liquid culture. The ability to grow on solid medium broadens the use of *S*. *marinoi* as a molecular model, where agar is routinely used for various purposes (growth, selection, storage). The ability to quantify colony growth opens the way for high throughput, automated or semi-automated phenotyping solutions.

## Methods

### Strains and culture conditions

*S*. *marinoi* strains GF0410J, HakH, and RO5AC were obtained from the culture collection GUMACC, (https://marine.gu.se/english/research/marine-biology/algal-bankGothenburg University Marine Algal Culture Collection) University of Gothenburg, Sweden. Cells were grown in filtered and autoclaved natural sea water (SW) with the salinity adjusted to 26 practical salinity units (psu), enriched with f/2 nutrients^28^ and 0.11 mM Na_2_SiO_3_ (Si). The strains were pre-cultured in 50 ml cell culture flasks (Sarstedt) with 40 ml of f/2 + Si medium at 16 °C, an irradiance of 50 µ mol photons m^−2^ s^−1^, and a photo-cycle 12Light:12Dark h for five to six days (late exponential growth phase) prior to the experiment start. Solid medium culturing was performed on 50 ml of 2% agar containing SW (salinity 26 and 10 psu) supplied with f/2 + Si at the same concentrations as for the liquid cultures. Agar was mixed with SW and dissolved by autoclaving; the nutrients and Si were added after the agar cooled to 60 °C, mixed by steering and poured into sterile rectangular plastic plates (Singer Instruments) under sterile conditions. The experimental cultures, in both types of culturing medium (solid and liquid), were tested at two different levels of salinity (26 and 10 psu) and temperature (10 °C and 16 °C). All microscopic observations were performed on an inverted light microscope (Axiovert 135, Zeiss); Dino-Eye eyepiece camera (Dino-Lite Digital Microscope) was used for cell imaging.

### Growth measurements and data analysis: liquid cultures

The cell abundances of the exponentially growing test strains were estimated using Sedgewick-Rafter cell counting chambers (Wildlife Supply Company) prior to the start of the experiment. Concentrated cultures were diluted in 40 ml of fresh f/2 + Si growth medium to 2 × 10^3^ cells ml^−1^ in three replicates per strain for each growth condition. Cell densities in two of the replicate bottles in each sample set were estimated daily for the first 15 days, and then once every alternate day during the following week. This was done using *in vivo* chlorophyll *a* fluorescence measurements (excitation wavelength: 425 nm, emission detection: 680 nm; Varioscan TM Flash Multimode Reader, ThermoScientific).

### Experimental setup and data acquisition: agar plates

The test *Skeletonema* strains were arranged on the agar surface in a fixed grid, matching the positions of well-centers of a standard 96-well plate, following the pattern in Supplementary Figure S1. Approximately 2 × 10^4^ cells per colony were transferred from the liquid-medium cultures in a 2 µl droplet using a multichannel pipette. In total, eight agar plates were prepared: two replicate plates for each of the four experimental conditions (salinity: 26 and 10 psu, temperature: 10 and 16 °C). All plates were imaged once per day for a period of 14 days using Canon EOS 70D with the following settings: F-stop - f/13, exposure time − 1/15 sec, ISO-speed - ISO-100, focal length − 42 mm. The camera was positioned at a fixed distance above the imaging surface, and was maintained in that position throughout the experiment. During imaging, agar plates were back-lit by a LED transluminator tablet and their lids were removed. The position of the light tablet and the plates on it were also maintained fixed in relation to the camera’s field of view for the duration of the experiment. The images were acquired and stored in both raw (CR2) and compressed (JPG) data formats. Only the raw images were used for the downstream analyses.

### Image processing

The image processing pipeline, detailed below, is illustrated in Supplementary Figure S3. Each image included four agar plates arranged in a custom-made acrylic glass fixture, containing key-point markers and a gray scale calibration strip (described in^1^). The images in a stack (a time series) were first aligned using linear registration^29^. The key-point markers of the fixture were identified on each image using template matching, and their locations were used to calculate the position of the calibration strip. The gray value scales were equalized across the image stack using histogram matching, where matching functions were calculated from the calibration strips using the last image of the stack as the reference. The stack was then split into four sub-stacks, one sub-stack for each plate. For each sub-stack, the colony grid was identified using manual placement of a grid of circles, adjusting the grid spacing and disc radius so that each colony was covered. With the colony bounds identified by this grid of circles, each sub-stack was cut into small squares to produce a stack of small images for each colony. For each colony stack, the extent of the colony was identified using the pixel-wise mean image. The mean image was first smoothed using median filtering to remove noise pixels, and then the colony area was segmented from background with Otsu’s method^30^. The inter-quantile mean of the background values was subtracted from the mean of the colony values, and this number was used as the value for each colony. This process pipeline produced a single value per time point for each of the 96 colonies on each plate of the experiment.

### Quantitative phenotypes

Each individual growth series was first cubic spline smoothed^31^, and then the three phenotypes were computed. The growth efficiency, or yield, was defined as the maximum value of the series. The maximal doubling rate (growth rate) was defined as maximal increase rate in log2 scale. This was computed from stepwise differences divided by step lengths, and, to avoid spurious rates, the maximum of the differences was discarded and the three next highest differences were averaged. The third phenotype was the time to the beginning of exponential growth phase, or lag. To estimate it we fitted the Gompertz growth curve model^32^ to each series. To be able to fit the model each series was first truncated before potential crash point (onset of the decline phase). We defined the crash point as the time after the maximum of a once more cubic spline smoothed series. Whenever the maximum was at the end of the series (9.5% of the curves), the maximum value was appended six times to end, in order to emulate a plateau phase and to improve the estimation of the model parameters (see Supplementary Fig. S4). The model was fitted using least squares minimization. The estimated delay-parameter of the model was taken as the lag.

### Statistical analysis

Colonies with extremely low ln-yield (<−4) were removed as dead colonies (20 out of 768, or 1.5%). We analyzed the phenotypes using linear regression modeling. The yield was analyzed in log scale, while rate and lag were analyzed in their original scale. To account for variations in the strains we included the interaction terms between treatments and strains in the model. No systematic deviation from normality was detected in the residuals so model fitness was deemed satisfactory. All analyses were run in the R-software^33^.

### Data availability

All data generated or analysed during this study are included in this article and its Supplementary Information file. Raw data are available from the corresponding author on request.

## Electronic supplementary material

Supplementary Information
