# Supplementary material for: Growth of a common planktonic diatom quantified using solid medium culturing
Source: Sci Rep. 2018 Jun 27;8:9757. doi: 10.1038/s41598-018-28129-y (PMC6021373; doi:10.1038/s41598-018-28129-y)
Supplement: Supplementary file 1 — Supplementary Information [file 41598_2018_28129_MOESM1_ESM.pdf]

Growth of a common planktonic diatom quantified using solid medium culturing

Olga Kourtchenko, Tuomas Rajala, Anna Godhe

Supplemental Information

Table S1. Change of model fit when a term is removed, as measured by AIC and residual sums of squares F-test

Salinity $*$ Temperature $*$ Strain + Replication, **AIC**	Yield -1652		Rate -2853		Lag 371	
**Change to AIC when term is removed**	**dAIC**	**Pr(>F)**	**dAIC**	**Pr(>F)**	**dAIC**	**Pr(>F)**
Salinity	38	<0.01	34	<0.01	291	<0.01
Temperature	-2	0.71	69	<0.01	392	<0.01
Strain	472	<0.01	149	<0.01	32	<0.01
Replication	2	0.06	2	0.05	0	0.2
Salinity & Temperature	11	<0.01	13	<0.01	114	<0.01
Salinity & Strain	39	<0.01	9	<0.01	-4	0.95
Temperature & Strain	2	0.05	14	<0.01	3	0.03
Salinity & Temperature & Strain	14	<0.01	-2	0.36	-3	0.63

a

	1	2	3	4	5	6	7	8	9	10	11	12
A	GF0410J	RO5AC	HakH	GF0410J	RO5AC	HakH	GF0410J	RO5AC	HakH	GF0410J	RO5AC	HakH
B	HakH	GF0410J	RO5AC	HakH	GF0410J	RO5AC	HakH	GF0410J	RO5AC	HakH	GF0410J	RO5AC
C	RO5AC	HakH	GF0410J	RO5AC	HakH	GF0410J	RO5AC	HakH	GF0410J	RO5AC	HakH	GF0410J
D	GF0410J	RO5AC	HakH	GF0410J	RO5AC	HakH	GF0410J	RO5AC	HakH	GF0410J	RO5AC	HakH
E	HakH	GF0410J	RO5AC	HakH	GF0410J	RO5AC	HakH	GF0410J	RO5AC	HakH	GF0410J	RO5AC
F	RO5AC	HakH	GF0410J	RO5AC	HakH	GF0410J	RO5AC	HakH	GF0410J	RO5AC	HakH	GF0410J
G	GF0410J	RO5AC	HakH	GF0410J	RO5AC	HakH	GF0410J	RO5AC	HakH	GF0410J	RO5AC	HakH
H	HakH	GF0410J	RO5AC	HakH	GF0410J	RO5AC	HakH	GF0410J	RO5AC	HakH	GF0410J	RO5AC

b

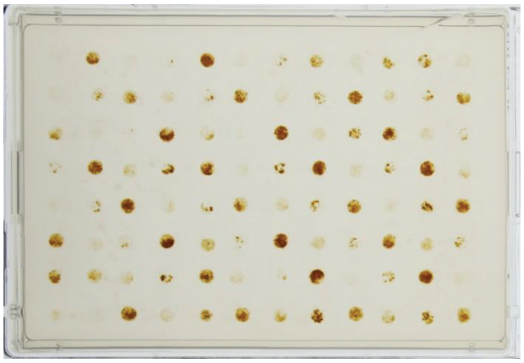

Figure S1. The layout of the strains in a 96-well plate format, with different colors representing different strains in the experiment. All instances of one strain are technical replicates of the same starting culture (**a**). An example image of the experimental solid medium plate in mid experiment (**b**).

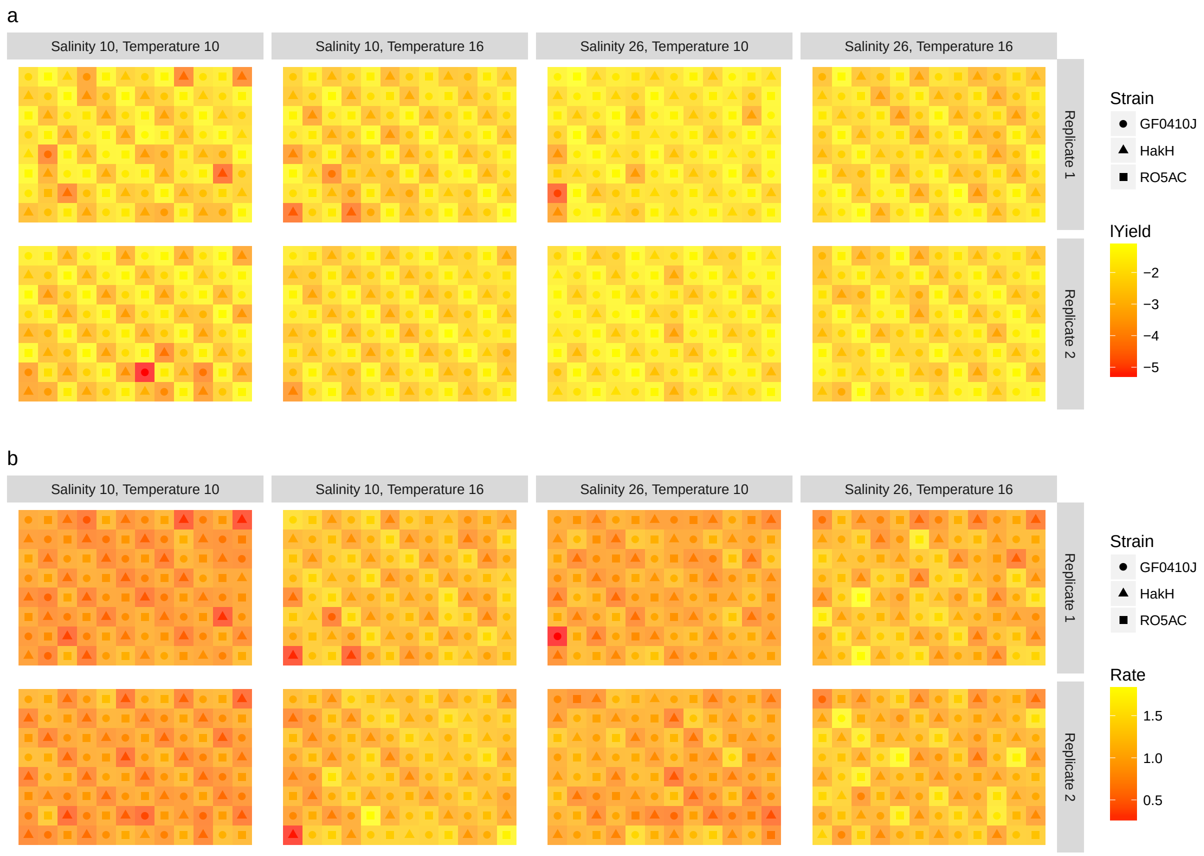

Figure S2. Heat-maps showing the distribution of the growth phenotypes values (ln of the maximum yield (**a**); growth rate (**b**)) of three *S. marinoi* strains (GF0410J - circle, HakH - triangle, RO5AC - square) over the surface of the solid medium plates under the respective treatment conditions. The color scale is yellow-to-red gradient, where yellow represents the highest observed values, and red - the lowest.

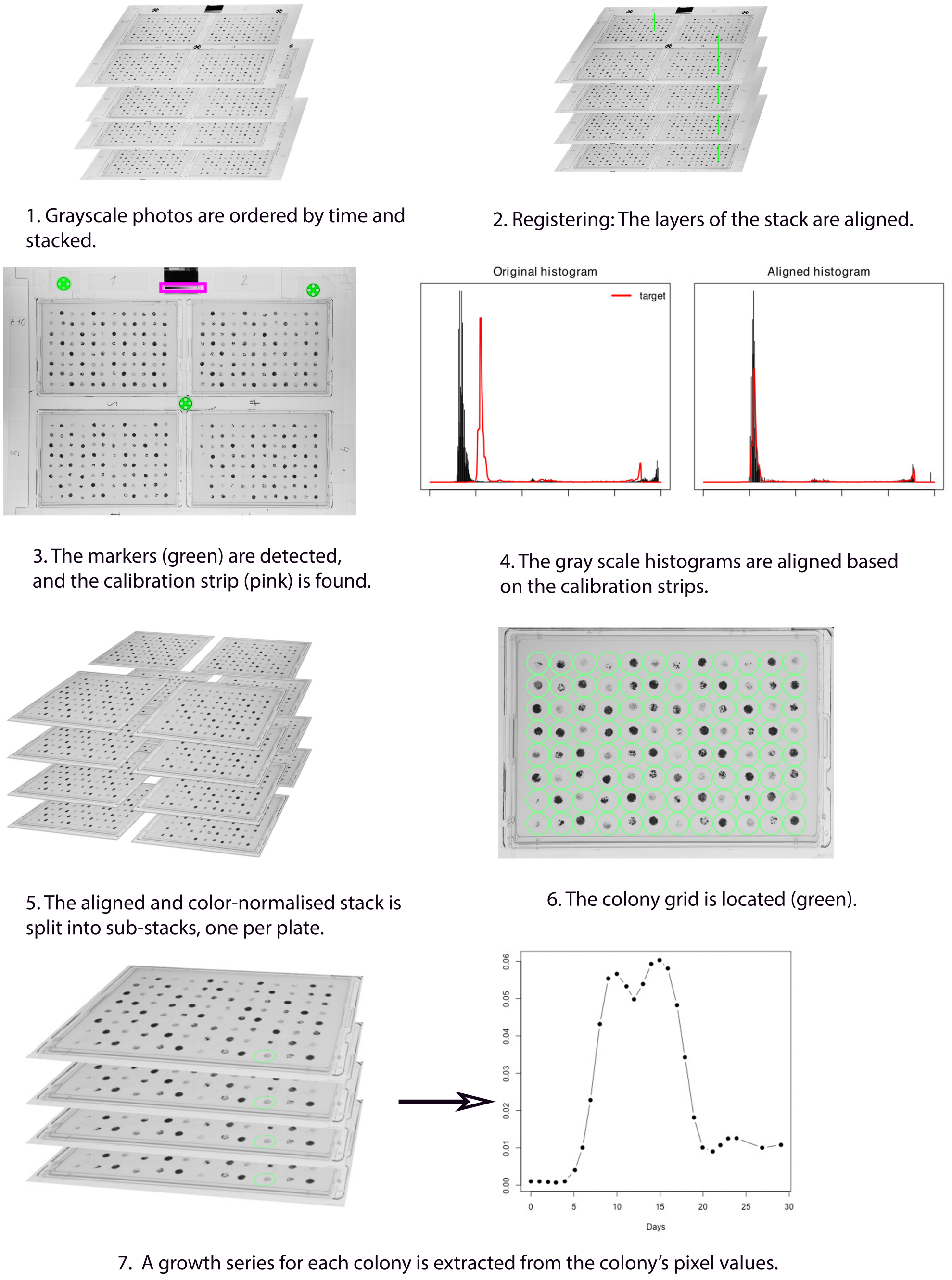

Figure S3. Image processing pipeline.

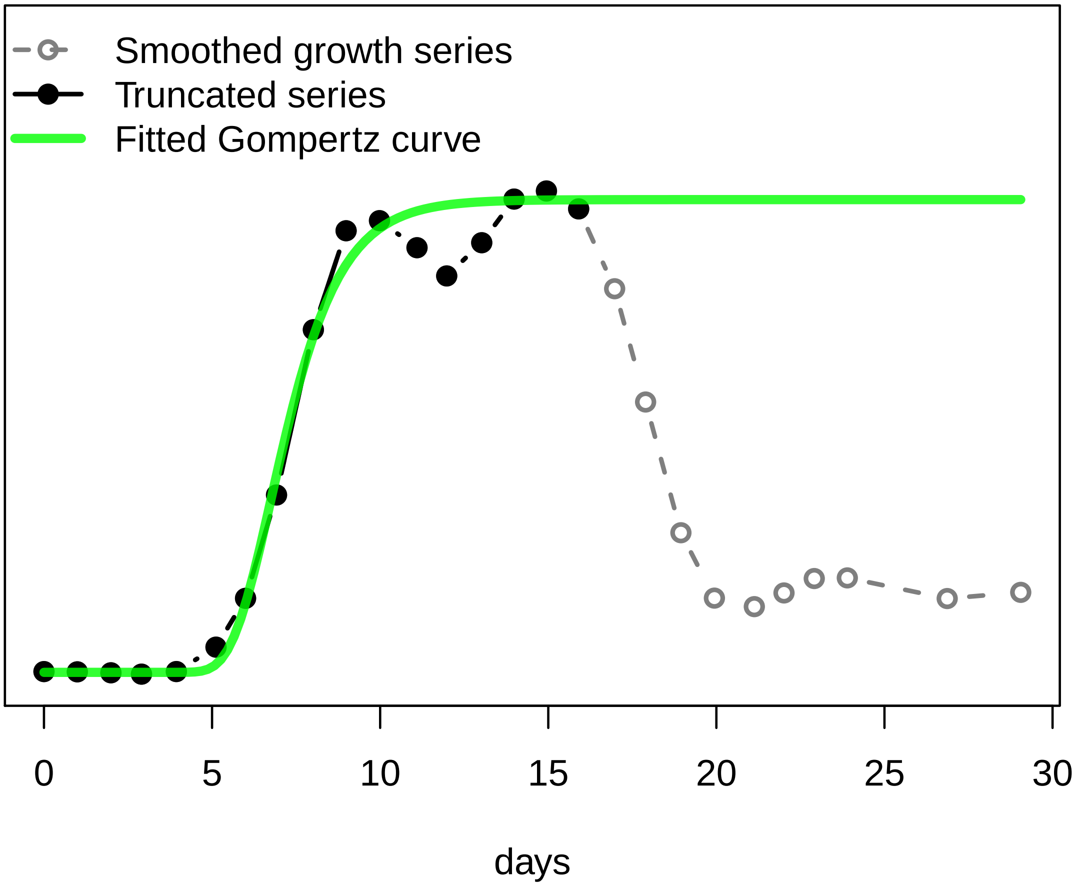

Figure S4. An example of growth curve processing.
